# A socioecological approach to understanding and positively affecting the intersectionality between disability, race and ethnicity, climate change, and rehabilitation outcomes: A scoping review

**DOI:** 10.1002/pmrj.13401

**Published:** 2025-06-21

**Authors:** Prateek Grover, Monica Verduzco‐Gutierrez, Thiru Annaswamy

**Affiliations:** ^1^ Department of Physical Medicine and Rehabilitation Penn State College of Medicine Hershey Pennsylvania USA; ^2^ Department of Rehabilitation Medicine University of Texas Health Science Center at San Antonio San Antonio Texas USA

## Abstract

**Background:**

Health care outcomes for people with disability may be disproportionately affected by climate change through multiple interlinked factors, which are not well understood.

**Objective:**

With use of scoping review methodology, this study aimed to model this intersectionality using socioecological (SE) levels to connect person‐level rehabilitation diagnoses with systems/policy‐level climate change and use this model to identify multilevel factors, rehabilitation outcomes, and responsive strategies from literature.

**Methods:**

A scoping review of literature was conducted using Preferred Reporting Items for Systematic Reviews and Meta‐Analyses Extension for Scoping Reviews methodology from three databases (PubMed Medline, Ovid Medline, CINAHL) using combinations of keywords (climate change), (rehabilitation), (disability), and (race). Logic and SE models were combined to model this intersectionality and create review forms that were used to abstract data. Common themes were collated (results), and additional experiential insight was added to provide contextual relevance (discussion).

**Results:**

Of 32 deduplicated articles, 11 met inclusion criteria for qualitative analysis. Rehabilitation outcomes included physical, economic, mental, cognitive, and mortality (person level); rehabilitation services disruption, medical supply delay, emergency capacity overwhelmed (organizational level); and disabled environment (community level). Responsive strategies included education, backup supplies, planning, social support/utility registration (person level); competency assessment/training, physical medicine and rehabilitation physicians (PM&R) assisting patient in planning, providing pre‐/postevent services, and establishing cross‐coverage (interpersonal level); telerehabilitation, energy/resources conservation, PM&R inclusion in disaster mitigation planning (organization level); building accessible/resilient infrastructure, evidence‐based practice guidelines through professional organizations (community level); and research funding, utility companies prioritizing power, and patients/providers included in planning (system/policy level).

**Discussion:**

Climate change impact on rehabilitation diagnoses such as spinal cord injury and limb loss, as well as intersectionality with rehabilitation outcomes and identified responsive strategies, has been comprehensively modeled using SE levels. Race is not a commonly identified factor.

**Conclusion:**

PM&R physicians can play a vital role in this intersectionality of disability, climate change, and rehabilitation outcomes.

## INTRODUCTION

Climate change profoundly affects public health.^1^ National public health agencies such as the Centers for Disease Control and Prevention report consequences related to communicable and noncommunicable diseases, food insecurity, and mental health (https://www.cdc.gov/climate-health/php/effects/index.html). Per the World Health Organization factsheet on climate change, a spectrum of “vulnerability factors,” such as gender, health system capacity, and sociopolitical environment (https://www.who.int/news‐room/fact‐sheets/detail/climate‐change‐and‐health), interact with environmental hazards and exposure to determine health risk. This impact is especially pronounced for people with disabilities, as related impairments, activity limitations, and participation restrictions could further increase their vulnerability. Examples of patient populations at increased risk include those with spinal cord injury (SCI)^2^, ^3^ and multiple sclerosis (MS).^4^ Although it is recognized that factors such as sociodemographic determinants, access to local health care, and climate change‐related policies can independently contribute to worse rehabilitation outcomes, their combined impact on outcomes, which can be considered a form of intersectionality, is not well studied. Additionally, within the field of physical medicine and rehabilitation (PM&R), the topic of climate change and health is not included in the standard residency educational curriculum. PM&R physicians and other rehabilitation professionals would benefit from a better understanding of how climate change affects the health of the patient populations they treat, what they can do to educate their patients, how to anticipate and minimize adverse impacts, and how to collaborate with administrators at their organization and advocate for responsive policies through their professional organizations.

To address this knowledge gap in the intersectionality between disability, race, climate change, and rehabilitation outcomes and to identify associated factors, relevant outcomes, and responsive strategies, logic models using the socioecological levels framework (SE) and comprehensive multilevel perspectives can be used. These models help create links between what we know (input construct), what can be done (output construct), and what is the impact (outcomes construct). The SE is a well‐accepted theoretical framework used in public health and medicine, including applications such as limb loss rehabilitation.^5^ It typically includes four to six hierarchical levels, including person, interpersonal, organization, community, system, and policy. Applying this standard multilevel approach to understanding the impact of climate change on rehabilitation outcomes for people with disabilities, and with the additional influence of race, is relatively novel and lends itself to a scoping review to refine research questions based on variables such as type of climate change, sociodemographics, rehabilitation diagnoses, stakeholder roles, outcomes, and policies.

The objective of this scoping review was to use the SE framework to model this intersectionality to connect person‐level rehabilitation diagnoses with systems‐/policy‐level climate change and use this model to identify multilevel factors, rehabilitation outcomes, and responsive strategies reported in the literature.

## METHODS

**FIGURE 1:**
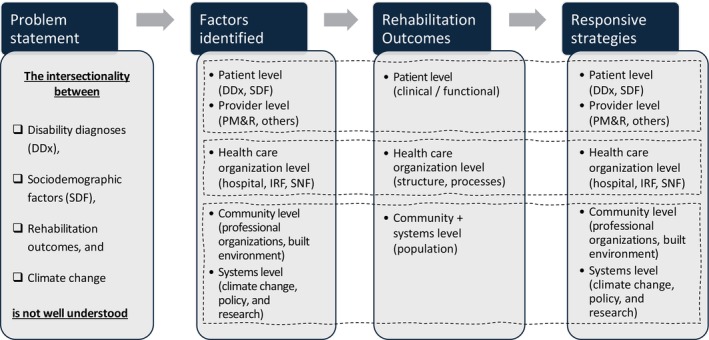
Logic model of intersectionality between disability, race, climate change, and rehabilitation outcomes. IRF, inpatient rehabilitation facility; PM&R, physical medicine and rehabilitation; SNF, skilled nursing facility.

**FIGURE 2:**
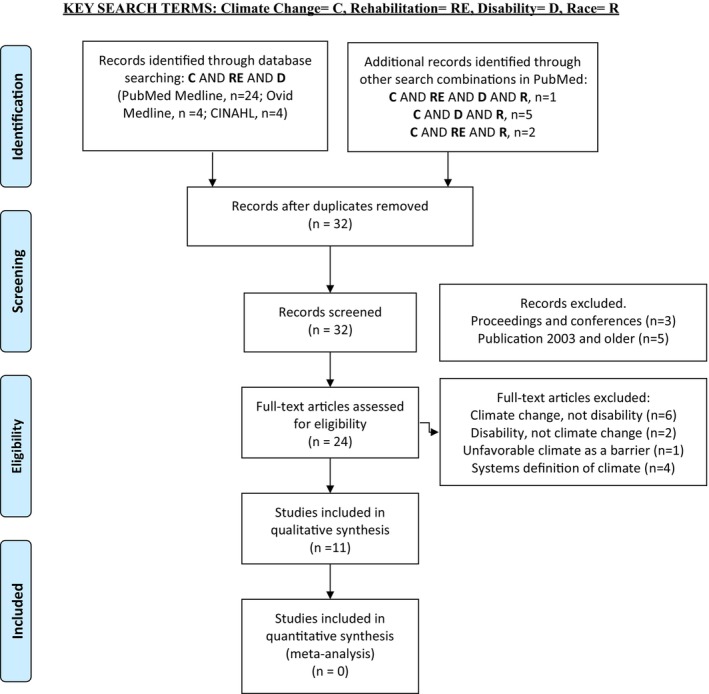
PRISMA‐ScR scoping review methodology for article selection. PRISMA‐SCR, Preferred Reporting Items for Systematic Reviews and Meta‐Analyses Extension for Scoping Reviews.

Logic models were used to create a framework with four linked constructs: limited understanding of intersectionality (problem statement) with factors identified (input), rehabilitation outcomes (outcomes), and responsive strategies (output). This was then combined with SE model levels to add multilevel perspectives (patient, provider, health care organization, community, systems) to the logic model constructs (Figure 1). This logic‐SE model framework was then used to create data review forms (Table 1). A scoping review of literature was then conducted by one author using Preferred Reporting Items for Systematic Reviews and Meta‐Analyses Extension for Scoping Reviews (PRISMaScr) methodology (Figure 2).^6^

**TABLE 1:** Logic model‐based data extraction tables for factors identified, outcomes/impact, and responsive strategies.

Data extraction form					
Factors identified					
Socioecological level	Person	Provider	Health care organization	Community	Health system
*Variables category examples*	*Disability diagnoses, sociodemographic factors*	*PM&R, therapy, primary care*	*Hospital, inpatient rehabilitation facility, skilled nursing facility*	*Professional organizations, built environment*	*Climate change, policies, research*
Linday et al., 2022	X	X	X	X	X
Taylor et al., 2022	X	X	X	X	X
Batten et al., 2020	X			X	X
Issac et al., 2023	X	X	X	X	X
Flores et al., 2020	X			X	X
Shapiro et al., 2020	X	X	X	X	X
Alexander M, 2020	X	X		X	X
Aryankhesal A, 2018	X	X		X	X
Gronlund CJ, 2014	X			X	X
Alexander et al., 2019	X	X	X	X	X
Alexander et al., 2019	X	X	X		X

Rehabilitation outcomes			
Socioecological level	Person	Health care organization	Community + health system
*Variables category examples*	*Clinical/functional*	*Process*	*Population*
Linday et al., 2022	X	X	X
Taylor et al., 2022	X	X	X
Batten et al., 2020	X		
Issac et al., 2023	X	X	X
Flores et al., 2020	X	X	
Shapiro et al., 2020	X		
Alexander M, 2020	X		X
Aryankhesal A, 2018	X		X
Gronlund CJ, 2014	X		
Alexander et al., 2019	X	X	X
Alexander et al., 2019	X	X	X

Responsive strategies					
Socioecological level	Person	Provider	Health care organization	Community	Health system
*Variables category examples*	*Disability diagnoses, sociodemographic factors*	*PM&R, therapy, primary care*	*Hospital, inpatient rehabilitation facility, skilled nursing facility*	*Professional organizations, built environment*	*Climate change, policies, research*
Linday et al., 2022	X	X	X	X	X
Taylor et al., 2022	X	X	X	X	X
Batten et al., 2020	X	X	X	X	X
Issac et al., 2023	X	X	X	X	X
Flores et al., 2020					X
Shapiro et al., 2020	X	X		X	X
Alexander M, 2020	X	X	X	X	X
Aryankhesal A, 2018	X	X		X	X
Gronlund CJ, 2014	X			X	X
Alexander et al., 2019	X	X	X	X	X
Alexander et al., 2019		X	X		X

Abbreviation: PM&R, physical medicine and rehabilitation.

The study review forms were then piloted and used by three authors to abstract data from articles. Common themes were then collated and discussed. Additional insight from current events and activities was added by the authors to provide contextual relevance.

### Eligibility criteria

No limits were set on the search, consistent with a scoping review. Detailed methodology for final article selection has been presented in the PRISMAScr diagram (Figure 2) and discussed in Selection of sources of evidence.

### Sources of evidence

PubMed Medline, Ovid Medline, CINAHL.

### Search strategy

The search strategies used the combination of keywords (climate change) AND (rehabilitation) AND (disability) in all three databases. Because few nonduplicative and relevant articles resulted from databases other than PubMed, other search combinations were used in PubMed only and included (climate change) AND (rehabilitation) AND (disability) AND (race); (climate change) AND (rehabilitation) AND (race); and (climate change) AND (disability) AND (race) (Figure 2).

### Charting methods

Study review forms were created for each of the three domains (factors identified, rehabilitation outcomes, and responsive interventions), using five SE levels (patient, provider, organization, community, and systems). These forms were piloted by two authors with one article each. The revised forms (Table 1) were then completed by all authors independently for an almost equal number of articles.

### Data items

Variable categories for both factors identified and responsive strategy domains included disability diagnosis and sociodemographic factors at the patient level; PM&R and other specialties at the provider level; hospital, inpatient rehabilitation facility (IRF), and skilled nursing facility (SNF) at the organization level; professional organizations and built environment at the community level; and climate change, policy, and research at the systems level. For the outcomes domain, variable categories included clinical and functional outcomes at the patient level, structure and processes at the health care organization level, and population outcomes at the community and systems levels.

### Synthesis of results

Results from the forms were collated and categorized by one reviewer and examined with the other reviewers to create summary recommendations that are further addressed in the Discussion section.

## RESULTS

### Selection of sources of evidence

A total of 40 articles resulted from the search, from which 8 duplicate records were deleted. Of the 32 remaining articles, eight articles were excluded proceedings and conferences [*n* = 3] and publication dating from 2003 and older [*n* = 5]). Twenty‐four articles were selected for full‐text review, and of these 13 articles were excluded for the following reasons: climate change, not disability focused (*n* = 6); disability, not climate change focused (*n* = 2); unfavorable climate as a barrier, no focus on change (*n* = 1); systems definition of climate (*n* = 4). This resulted in a final set of 11 articles that were used for qualitative synthesis.^2^, ^3^, ^4^, ^7^, ^8^, ^9^, ^10^, ^11^, ^12^, ^13^, ^14^ (Figure 2).

### Characteristics and results of sources of evidence

Study type included the following: reviews (*n* = 3),^12^, ^13^, ^14^ survey patients (*n* = 2),^9^, ^10^ survey professionals (*n* = 1),^8^ survey general population (*n* = 1),^10^ opinion/perspective/position (*n* = 4).^2^, ^3^, ^4^, ^7^ A majority of the studies were from the United States (*n* = 8), with one each from Australia,^10^ Canada,^14^ and Iran.^9^ Seven articles were published in the last 5 years.^3^, ^4^, ^7^, ^10^, ^11^, ^13^, ^14^ (Table 2).

**TABLE 2:** Characteristics of sources of evidence, rehabilitation conditions and impairments described.

S No.	Articles	Study type^c^	Sample size^a^	Country of origin	Diagnosis					Impairment domains			
					LL	ALS	MS	SCI	CVA	Physical/mobility	Cognition	Vision	Misc
1	Lindsay et al., 2022	1		Canada			X	X	X	X	X	X	Various
2	Taylor et al., 2022	5		United States		X	X	X	X	X			General
3	Batten et al., 2020	2	14	Australia	X					X			
4	Issac et al., 2023	1		United States					X	X	X		
5	Flores et al., 2020	4	403	United States						X	X		Evacuation disability^b^
6	Shapiro et al., 2020	5		United States				X		X			
7	Alexander M, 2020	5		United States				X		X			COVID‐19 related challenges
8	Aryankhesal et al., 2018	2	12	Iran						X			
9	Gronlund CJ, 2014	1		United States						X	X		Mental health
10	Alexander et al., 2019	3	125	United States				X		X			
11	Alexander et al., 2019	5		United States				X		X			

Abbreviations: ALS, amyotrophic lateral sclerosis; CVA, cerebrovascular accident/stroke; LL, limb loss/amputation; MS, multiple sclerosis; SCI, spinal cord injury; S No, study number.

^a^ Sample size is reported for survey studies (patients, professionals, general population).

^b^ Self‐identified physical, mental, or cognitive condition that required special assistance for evacuation.

^c^ Study type: review = 1, survey patients = 2, survey professionals = 3, survey general population = 4, opinion/perspective/position = 5.

### Results of individual sources of evidence and synthesis of results

#### FACTORS IDENTIFIED

##### System level

###### Categorization of climate change

Categories of climate change described in the articles included hurricane/typhoon (*n* = 5),^2^, ^3^, ^11^, ^13^, ^14^ temperature—hot and cold (*n* = 5),^2^, ^8^, ^10^, ^12^, ^14^ flood and drought (*n* = 3),^2^, ^8^, ^14^ wildfire and air pollution (*n* = 3),^2^, ^4^, ^8^ earthquake (*n* = 1),^9^ and infectious disease (*n* = 1).^4^

##### Patient level

###### Rehabilitation diagnoses and comorbidities

included SCI (*n* = 5),^2^, ^3^, ^4^, ^7^, ^8^ MS (n = 1),^4^ amyotrophic lateral sclerosis (ALS) (*n* = 1),^4^ stroke (*n* = 1),^13^ amputation (*n* = 1),^10^ and cardiorespiratory conditions (*n* = 1).^12^ Mental health diagnoses (*n* = 2)^12^, ^13^ and COVID (n = 1)^7^ were addressed as well.

###### Impairments and activity limitations

described included physical/mobility (*n* = 6),^2^, ^4^, ^9^, ^12^, ^13^, ^14^ daily activities (*n* = 1),^13^ cognition (*n* = 3),^12^, ^13^, ^14^ swallowing (*n* = 1),^13^ vision (*n* = 2),^13^, ^14^ and sensory/thermoregulation (*n* = 2).^4^, ^14^ *Sociodemographic factors* were described by five articles.^7^, ^8^, ^9^, ^11^, ^12^ These included race and ethnicity, gender, literacy, education, work, income, immigration, and neighborhood. Other factors included low resilience.^4^, ^14^

##### Care provider level

Two recurring themes include lack of knowledge about managing special needs for people with disabilities among clinical care providers,^3^, ^14^ and the responsibility for PM&R physicians to ensureg pre‐event, intra‐event, and post‐event optimal management of rehabilitation conditions.^2^, ^3^, ^7^

##### Organization level

Two themes include pre‐event access to regular and emergency services, medications, and transportation,^3^, ^4^, ^8^, ^14^ and contribution of health care itself to climate change.^4^

##### Community + systems level

Housing conditions and less accessible neighborhoods in areas of higher disability are contributory.^8^, ^11^, ^12^, ^14^

#### REHABILITATION OUTCOMES

##### Patient level

Physical impact (*n* = 8)^3^, ^4^, ^7^, ^8^, ^9^, ^10^, ^13^, ^14^ is the most common outcome described, followed by economic (*n* = 4),^3^, ^7^, ^11^, ^14^ mental (*n* = 3),^11^, ^13^, ^14^ emotional (*n* = 3),^3^, ^13^, ^14^ and mortality (*n* = 2).^4^, ^12^

##### Organization level

Disruption of rehabilitation services (IRF, SNF, home health, outpatient therapy),^4^, ^13^, ^14^ medical supply delays,^13^ and emergency capacity overwhelmed (emergency medical services, nondisabled people seeking urgent care)^13^ are some common themes.

##### Community + systems level

Disabling environment^4^, ^13^, ^14^ has been described as the common theme by many articles.

#### RESPONSIVE STRATEGIES

##### Patient level

Strategies included education, backup supplies, plans (*n* = 9),^3^, ^4^, ^7^, ^8^, ^9^, ^10^, ^12^, ^13^, ^14^ and social support/utility registration (*n* = 5).^3^, ^7^, ^9^, ^13^, ^14^

##### Provider level

Strategies included competency assessment and training (n = 5),^2^, ^4^, ^7^, ^8^, ^14^ PM&R physicians helping patients plan, and provide/coordinate pre‐ and postevent services, as well as organize cross‐coverage (*n* = 4).^2^, ^3^, ^4^, ^7^

##### Organization level

Strategies included use of telerehabilitation, reduction of waste of energy and resources (*n* = 4),^2^, ^4^, ^7^, ^8^ inclusion of PM&R in disaster mitigation plans (*n* = 3),^3^, ^4^, ^7^ and improvement of rehabilitation services (*n* = 1).^14^

##### Community level

Strategies included building accessible and resilient infrastructure (*n* = 5),^7^, ^9^, ^10^, ^12^, ^14^ and dissemination of evidence‐based practice guidelines by professional organizations (*n* = 1).^4^

##### Systems level

Strategies included research funding and policies for coverage (*n* = 6),^2^, ^4^, ^7^, ^8^, ^10^, ^12^ utility companies prioritizing power using a standard registration process (*n* = 2),^3^, ^13^ and including patients and providers in planning (disability inclusiveness) (*n* = 5).^3^, ^4^, ^7^, ^10^, ^14^

## DISCUSSION

This scoping review is novel in approaching the intersectionality between disability, race, climate change, and rehabilitation outcomes by using implementation science principles, including a logic model and SE levels for modeling and extracting data from literature to identify not only multilevel factors and outcomes but also multilevel responsive strategies at the person, interpersonal, organization, community, and health system levels. The type of articles in this scoping review include surveys, reviews, and perspectives, with most being recent, indicating an accelerating focus on understanding the interplay between climate change and disability. A preponderance of articles (8 of 11) came from the United States, indicating a level of awareness within the U.S. PM&R community regarding climate change and disability. Definitive data on the magnitude of work being done and resources available will require further study. All articles described some factors, outcomes, and responsive strategies, indicating the solution‐oriented focus.

### FACTORS AND OUTCOMES

Persons with disabilities face barriers from climate‐related events that are unique to their primary and secondary medical diagnoses resulting in their specific set of impairments, activity limitations, and participation restrictions. In addition to these patient‐level factors, this review identified a multitude of other barriers and challenges at multiple SEs that warrant further discussion. Better conceptualization and characterization of these factors can facilitate future in‐depth investigation and identification of potential interventions and responsive strategies to mitigate the impact of these factors on the rehabilitation outcomes of persons with disabilities. All the associated factors identified in this scoping review are related to climate change‐related extreme events such as hurricanes, earthquakes, and floods. However, there is increasing emphasis on raising awareness and understanding of the subtle yet significant effects of climate change, such as gradually increasing temperatures, rise in sea levels, and other related phenomena. Further research is needed to better understand the effects of insidious climate change on vulnerable populations such as persons with disabilities, and this review makes a strong case in this regard.

#### Person level

This review discusses how medical diagnoses such as SCI, stroke, ALS, limb loss, cardiorespiratory illness, MS, psychological illness, and COVID‐19 (Table 2) affect rehabilitation outcomes during climate change events. Examples of person‐level factors that help illustrate this increased sensitivity of individuals to obstacles posed by the effect of climate change include mobility and daily‐activity challenges faced during disaster events such as hurricanes/typhoons, earthquakes, and floods; exacerbation of symptoms due to influence of extreme fluctuations in temperature such as extreme hot and cold waves, drought, famine, and pollution from wildfires and smog; and the effects of infectious disease pandemics. Other person‐level impairment and activity limitation domains that affect the outcomes–climate change relationship identified in this review include cognition, vision, thermoregulation, swallowing, and sensory perception (Table 2).

The review identifies social determinants of health such as race and ethnicity, gender, literacy, education, work, income, immigration, and neighborhood as important factors that intersected with persons with disabilities that compounded their vulnerabilities to climate change. The review also highlights low resilience, which is not always well recognized in disability literature. Better understanding of these person‐level factors unique to individual patients who are under the care of rehabilitation providers can enable these providers to better manage the effects of these factors on adverse rehabilitation outcomes during climate change influenced events.

#### Provider level

This scoping review hightlights inadequate training and lack of knowledge among rehabilitation providers and trainees about managing special needs for people with disabilities (Table 1). This review places the responsibility on PM&R physicians and rehabilitation providers to seek and acquire training so that they are better equipped to manage their patients with disabilities during pre‐event, intra‐event, and post‐event situations. Even though disaster preparedness training may be available at a generic level that is applicable to the population at large, there is a lack of training specific to the care of persons with disabling conditions that rehabilitation providers need proficiency in.

#### Organizational level

Findings in this review indicate that hospitals and health care facilities may not engage PM&R physicians and rehabilitation providers in disaster preparedness activities. As a result, the unique needs of persons with disabilities that may not be addressed adequately by such disaster preparedness activities may not be well represented by these organizations. This review highlights this unmet need and calls for specific interventions to address this gap.

#### Community level

This review calls out the need for the rehabilitation community to engage at the grassroots level with the communities of persons with disabilities to better understand their unmet needs at a community level. Without grassroots‐level organization, unique community‐level factors that may affect communities with large populations of persons with disabilities, such as assisted living or senior‐living communities, may not be optimally identified and addressed.

#### Systems level

This review highlights that policies, laws, and regulations that affect the health and rehabilitation outcomes of persons with disabilities as they pertain to effects of climate change are still evolving. The evolving political, legal, and regulatory landscape poses unique challenges because it limits the identification and application of responsive strategies needed to optimize the rehabilitation care of persons with disabilities impacted by climate change. Hence, continued advocacy at local, regional, and federal levels is vital to influence policy change that will have a positive impact on the care of persons with disabilities affected by climate change.

### RESPONSIVE STRATEGIES

This review underscores the significant influence of climate change on rehabilitation outcomes. Individuals with disabilities face barriers that jeopardize the delivery of optimal rehabilitation services during climate‐related events. A multitude of challenges at various levels of care across the rehabilitation continuum have been discussed and solutions at each SE level are warranted. In response to the identified challenges, a range of responsive strategies and potential interventions were found in this scoping review.

#### Person‐level interventions

These interventions focus on education and preparedness. There should be education seminars and training programs for individuals with disabilities on disaster preparedness, including creating individualized emergency plans and ensuring access to relevant information. The resources created should be tailored to their specific needs and limitations. Furthermore, facilitating social support networks and community networks is necessary. Examples include providing assistance with registering for utility priority services to ensure timely access to essential resources such as electricity for a person who requires ventilator support. Aryankhesal and colleagues discussed specific examples of safety needs during an earthquake including ensuring that persons with disabilities have their room secured prior to an earthquake to increase safety, especially should they be unable to go outside the room after an earthquake.^9^

#### Provider‐level interventions

These include conducting competency assessments and training programs for health care professionals, including PM&R physicians and rehabilitation professionals, to enhance knowledge and skills in managing the special needs of individuals with disabilities during climate‐related events. Engaging rehabilitation professionals in collaborative care planning with patients can optimize outcomes in the setting of a climate‐related event. There should be an emphasis on pre‐event, intra‐event, and postevent services and coordinating cross‐coverage plans. Moreover, this education should be provided at the undergraduate and graduate medical education levels.

#### Organizational‐level interventions

These include leveraging telerehabilitation technologies to deliver rehabilitation services and clinician consultations remotely.^7^ Speech therapy, psychological services, physiotherapy, and occupational therapy services can be delivered remotely. Telemedicine resources for PM&R physicians have also been developed and these can be easily adopted for remote care.^15^ Though telemedicine improves access to health care, barriers for persons with disabilities have been described. Nevertheless, it can help address challenges related to physical access, transportation, and infrastructure disruptions during climate‐related events. Also related to the organizational level is the need to advocate for the inclusion of rehabilitation professionals and services in disaster mitigation plans at community levels. This ensures the specific needs of individuals with disabilities are adequately addressed. Finally, we must implement strategies to reduce energy consumption and minimize resource waste in the health care sector. This contributes to environmental sustainability in the face of considerable climate change.

#### System‐ and policy‐level interventions

These are some of the most important and yet most challenging aspects. Advocating for increased research funding and policy support to address intersection between disability, race, climate change, and rehabilitation outcomes is needed now. Prioritizing these research initiatives and disability inclusiveness is of utmost importance for PM&R physicians.

### LIMITATIONS

Although data were extracted to the best of the authors' ability, it was not possible to completely represent all themes from all articles. It is likely there are more articles that describe the intersection of factors but that would likely be consistent with the scope of a systematic review.

## CONCLUSION

There is compelling evidence for the intersectionality between disability diagnoses, sociodemographic factors such as race, climate change, and rehabilitation outcomes. There are several multilevel strategies responsive to factors identified that can be used by PM&R to positively affect these rehabilitation outcomes. Recognizing and addressing these complexities are crucial for developing effective rehabilitation strategies and enhancing outcomes. Rehabilitation professions can play a vital role in improving public health in the face of climate change.

## DISCLOSURE

No disclosures related to this publication.

## Supporting information


Data S1
