# Supplementary material for: A socioecological approach to understanding and positively affecting the intersectionality between disability, race and ethnicity, climate change, and rehabilitation outcomes: A scoping review
Source: PM R. 2025 Jun 21;18(Suppl 1):S35–44. doi: 10.1002/pmrj.13401 (PMC12903836; doi:10.1002/pmrj.13401)
Supplement: Supplementary file 1 — Data S1 [file PMRJ-18-S35-s001.doc]

**APPENDIX**

**List of Articles included in the scoping review**

1. Alexander M. . Sustainability and spinal cord injury: attending to climate change and disability. Spinal Cord Ser Cases. 2019 Oct 21;5:88. doi: 10.1038/s41394-019-0232-6. eCollection 2019.PMID: 31700686 Free PMC article. No abstract available.
2. Alexander M, Alexander J, Arora M, Slocum C, Middleton J. A bellweather for climate change and disability: educational needs of rehabilitation professionals regarding disaster management and spinal cord injuries. Spinal Cord Ser Cases. 2019 Nov 15;5:94. doi: 10.1038/s41394-019-0239-z. eCollection 2019.PMID: 31754472 .
3. Alexander M. It's Corona Calling: Time for Telerehabilitation. J Frailty Sarcopenia Falls. 2020 Dec 1;5(4):86-88. doi: 10.22540/JFSF-05-086. eCollection 2020 Dec. PMID: 33283073
4. Aryankhesal A, Pakjouei S, Kamali M. Safety Needs of People With Disabilities During Earthquakes. Disaster Med Public Health Prep. 2018 Oct;12(5):615-621. doi: 10.1017/dmp.2017.121. Epub 2017 Oct 2.PMID: 28965529
5. Batten H, Lamont R, Kuys S, McPhail S, Mandrusiak A. What are the barriers and enablers that people with a lower limb amputation experience when walking in the community? Disabil Rehabil. 2020 Dec;42(24):3481-3487. doi: 10.1080/09638288.2019.1597177. Epub 2019 Apr 13.PMID: 30982367
6. Flores AB, Collins TW, Grineski SE, Chakraborty J. . Disparities in Health Effects and Access to Health Care Among Houston Area Residents After Hurricane Harvey. Public Health Rep. 2020 Jul/Aug;135(4):511-523. doi: 10.1177/0033354920930133. Epub 2020 Jun 15.PMID: 32539542
7. Gronlund CJ. . Racial and socioeconomic disparities in heat-related health effects and their mechanisms: a review. Curr Epidemiol Rep. 2014 Sep 1;1(3):165-173. doi: 10.1007/s40471-014-0014-4.PMID: 25512891
8. Issac S, Shultz JM, Espinel Z, Alvarez G, Shapiro LT. In the Cone of Concern: Preparing Stroke Survivors for Extreme Hurricanes..Am J Phys Med Rehabil. 2023 Feb 23. doi: 10.1097/PHM.0000000000002221. Online ahead of print.PMID: 36917040
9. Lindsay S, Hsu S, Ragunathan S, Lindsay The impact of climate change related extreme weather events on people with pre-existing disabilities and chronic conditions: a scoping review. J.Disabil Rehabil. 2022 Nov 25:1-21. doi: 10.1080/09638288.2022.2150328. Online ahead of print.PMID: 36426560
10. Shapiro LT, Gater DR Jr, Shultz JM. It is time to put hurricane preparedness on the radar for individuals living with spinal cord injury. Spinal Cord Ser Cases. 2020 May 11;6(1):34. doi: 10.1038/s41394-020-0282-9.PMID: 32393807 Review.
11. Taylor AL, Perret D, Morice K, Zafonte R, Skelton F, Rivers E, Alexander M. Climate Change and Physiatry: A Call to Proportional and Prospective Action. Am J Phys Med Rehabil. 2022 Oct 1;101(10):988-993. doi: 10.1097/PHM.0000000000002017. Epub 2022 Mar 29.PMID: 35363629
